# Relationship between Food Crushing and Oral Function in Older Adults Requiring Nursing Home Care: A Pilot Study

**DOI:** 10.3390/ijerph19063419

**Published:** 2022-03-14

**Authors:** Kanako Yamawaki, Takahiro Mori, Sakiko Itaki, Azusa Haruta, Chiho Takeda, Aya Hiraoka, Mariko Maruyama, Mineka Yoshikawa, Mitsuyoshi Yoshida, Kazuhiro Tsuga

**Affiliations:** 1 Departments of Advanced Prosthodontics, Graduate School of Biomedical and Health Sciences, Hiroshima University, Kasumi, 1-2-3, Minami-ku, Hiroshima 734-8553, Japan; takahiro-mori@hiroshima-u.ac.jp (T.M.); harutazusa@hiroshima-u.ac.jp (A.H.); chihotakeda@hiroshima-u.ac.jp (C.T.); sato-0903@hiroshima-u.ac.jp (A.H.); m-maruyama@hiroshima-u.ac.jp (M.M.); mineka@hiroshima-u.ac.jp (M.Y.); tsuga@hiroshima-u.ac.jp (K.T.); 2 PIA Nakamura Hospital, Tsuboi, 3-818-1, Saeki-ku, Hiroshima 731-5142, Japan; itamochi648@gmail.com; 3 Departments of Dentistry and Oral-Maxillofacial Surgery, School of Medicine, Fujita Health University, Dengakugakubo, 1-98, Kutsukake-cho, Toyoake 470-1192, Japan; mitsuyoshi.yoshida@fujita-hu.ac.jp

**Keywords:** diet modification, dysphagia, geriatric dentistry, geriatric nursing, mastication

## Abstract

We investigated how jelly is crushed and examined the relationship between tongue pressure and tongue food crushing ability among older adults requiring nursing home care. Seventy-two participants were instructed to freely crush the test foods soft jelly (SJ) and hard jelly (HJ). We visually evaluated the crushability of the test food and identified the intraoral tissues (active sites) used to crush the test food. The active sites were consistent for all participants for both SJ and HJ, and they included the maxillary and mandibular teeth in 41 participants, teeth and residual ridges in 15 participants, maxillary and mandibular residual ridges in 10 participants, and tongue and palate in six participants. Two participants failed to crush the SJ; the active sites in both participants were the tongue and palate. No participant using the tongue and palate as active sites could crush the HJ. Furthermore, 64 participants could crush the SJ and 23 could crush the HJ using the tongue and palate. The cutoff value of the tongue pressure for crushability of the HJ was 22.0 kPa. Assessing tongue pressure and intraoral active sites involved in food crushing could help determine an appropriate diet for older adults requiring nursing home care.

## 1. Introduction

Older adults who require caregiving find it difficult to visit dental clinics because of cognitive decline and reduced capacity with respect to activities of daily living (ADL) [1]. Consequently, these adults with declining dental health live in social withdrawal due to aesthetic changes, deterioration of quality of life, and reduced ability to communicate. Ingesting tangible foods is nutritious [2]; however, some people tend to eat less tangible food when oral health problems occur despite the possibility of retaining masticatory function. Some older adults that require nursing care may need to consume lower than optimal levels of tangible food because of restricted access to dental care. Oral function is complex, and it is difficult to evaluate occlusal force and masticatory function.

Older adults in nursing homes often have missing teeth and reduced oral function [3,4]. Some of them also have reduced cognitive function and missing teeth and may need to eat meals without wearing dentures [5,6,7]. People that do not wear dentures, despite the lack of occlusal contact between the remaining teeth, use the residual ridges or tongue to crush food. However, no previous study has examined the intraoral sites of food crushing in older adults requiring nursing care.

In Japan, choking is the most common cause of accident-related mortality, accounting for approximately 9000 deaths annually; approximately 90% of cases occur in adults aged ≥65 years [8]. Dementia and decreased oral function are risk factors for choking [9,10]. Therefore, assessing oral function, including food crushing ability, is required to determine the food form that could prevent choking in this group.

Recent studies have proposed a classification of modified diets for dysphagia, providing a list of suitable foods for people with reduced swallowing function [11]. A diet modified for dysphagia accounts for the hardness, adhesiveness, and cohesiveness of the food [12]. In particular, the hardness of the food is an important factor determining if an individual can crush it intraorally [13,14,15]. While food is primarily crushed using the remaining teeth and dentures, older adults with missing teeth who do not wear dentures may use the residual ridge and tongue to crush their food.

“Universal design food” has been proposed by the Japan Care Food Conference as a care food [16]. The classification of universal design foods includes foods labeled as “easily chewable (<5 × 10^5^ N/m²)”, “gum-mashable (<5 × 10^4^ N/m²)”, “tongue-mashable (<2 × 10^4^ N/m²)”, and “no need to chew (<5 × 10^3^ N/m²)”; food hardness is defined per category [17,18,19]. Many hospitals and facilities define these food forms and provide corresponding meals to older adults that require care [20]. However, the definitions of these foods are considered approximate clinical indicators, and there are no objective indicators in this context. Food crushing ability remains unclear in older adults; elucidating suitable food hardness on the basis of the intraoral condition of older adults requiring assistive care is required.

The tongue is one of the oral organs used to crush food, and a tongue–palate pressure test may help quantify an individual’s ability to crush food using the tongue [21]. This test involves placing a balloon-shaped pressure sensor at the front of the palate and recording the pressure between the tongue and palate as the tongue is pressed upward against the balloon [21,22]. This test is widely used to assess tongue function, impact of aging on tongue pressure [23,24], and the relationship between tongue pressure and dysphagia [25,26]. Although there have been reports on the relationship between masticatory function and tongue pressure in young people [27], no studies to date have examined the relationship between tongue pressure and tongue food crushing ability among older adults requiring care, including those with dementia.

In this study, we investigated the crushability of food in the oral cavity and the intraoral tissues used in free food crushing. Specifically, we aimed to elucidate the relationship between the pressure of the tongue and the crushability of the food with the tongue in older adults requiring nursing home care, using two types of test food. These findings may help establish a method of oral function assessment that could help determine the food form that can be safely administered to these patients.

## 2. Materials and Methods

### 2.1. Study Participants

Of 189 older adults living in an assisted living medical institution and receiving regular dental checkups from November 2017 to October 2018, 72 participants who met the following criteria were selected: oral ingestion of three meals a day; ability to understand and follow simple instructions, such as “do not swallow the test food”; no acute systemic or dental symptoms; consent to study participation provided by participant or next of kin.

Written informed consent was obtained from all participants. This study was approved by the Nakamura Hospital Ethics Committee (Approval No.: D-18).

### 2.2. Test Foods

A soft jelly (SJ) as hard as rice porridge or scrambled egg (plunger diameter 40 mm, height 15 mm, compression speed 10 mm/s, clearance 5 mm, height of the first compression peak when compressed twice at a constant speed) [28] at approximately 1.51 × 10^4^ N/m² (Oishikusen’i^®^, peach flavor, House Foods Corporation, Ltd., Tokyo, Japan) and a hard jelly (HJ) as hard as soft rice or rolled egg at approximately 2.73 × 10^4^ N/m² (Minijelly^®^, peach flavor, Nisshin OilliO Group, Ltd., Tokyo, Japan) were each cut into pieces of 30 mm (length) × 20 mm (width) × 6 mm (thickness). The hardness of SJ corresponds to the “tongue-mashable” category of universal design food, and the hardness of HJ corresponds to the “gum-mashable” category [16,17,18,19]. The size of the jelly was referred to the sliced jelly used to start swallowing training [29].

### 2.3. Test 1 (Free Crush)

The participants were instructed to crush the jelly freely in the mouth without swallowing it and to open the mouth after the jelly had been crushed. The participants using dentures were tested while wearing dentures as they would during routine meals. We observed by visual examination if the test food had been crushed or not (crushability), as well as the intraoral tissues (active site(s)) used to exert food crushing force. The test was first conducted using SJ, and, after thorough rinsing, the test was repeated using HJ.

#### 2.3.1. Crushability

**Figure 1:**
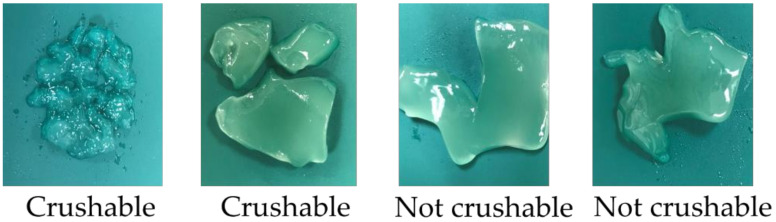
Criteria to determine the crushability of test food.

Whether the test food had been crushed or not was ascertained on the basis of the splitting of the test food, with two or more splits suggesting that the food was “crushable” and any other result indicating that the food was “not crushable” (Figure 1).

#### 2.3.2. Active Sites

The assessment of active sites was performed by visual examination by two examiners and a medical interview with the participant. Visual examination was based on their mandibular movement [30] and the intraoral sites the test food stayed on, regardless of food crushability, when the participant completed the crushing movements and opened the mouth. The definitions and classification of the active sites are presented in Table 1. If no clear determination could be made about the active sites, we repeated the test and confirmed whether the examiners’ visual assessment findings were consistent with the active sites reported by the participants.

### 2.4. Test 2 (Tongue Crush)

**Figure 2:**
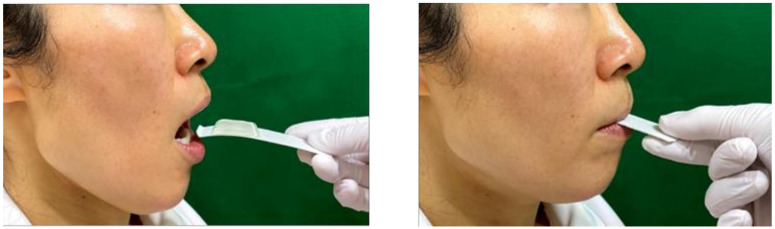
Tongue crush assessment.

To investigate the crushability of food by the tongue, we placed the test food on a 24 mm × 85 mm cardboard spoon and instructed the participants to press and crush the jelly with their tongue (Figure 2). As such, we evaluated the crushability of the two types of test foods when the participant raised their tongue upward with the cardboard spoon inserted into their oral cavity. Tongue pressure was measured three times using a tongue pressure measurement device (TPM-01^®^, JMS, Hiroshima), and the average value was recorded [21,22].

The criteria used to determine the crushability of test food were comparable to those used in Test 1 (see Figure 1).

### 2.5. Other Parameters of Assessment

The use of dentures, presence or absence of occlusal support from the remaining teeth or dentures, number of remaining teeth, food form (normal food, chopped food, modified diet for dysphagia), ADL (ambulatory, wheelchair use, bedridden), nutritional status, and cognitive function were assessed. Nutritional status was evaluated using the Mini Nutritional Assessment-Short Form (MNA-SF) [31], with a score of 12–14 points indicating “good” nutrition status, a score of 8–11 points indicating “at risk” status, and a score of <7 points indicating “malnutrition”. Cognitive function was evaluated using the Mini-Mental State Examination (MMSE) [32].

### 2.6. Statistical Analyses

All statistical analyses were performed using BellCurve for Excel version 3.00 (Social Survey Research Information Co., Ltd., Tokyo, Japan). To compare age, MMSE scores, and tongue pressure values per active site, we used the Tukey–Kramer test for multiple comparisons after one-way analysis of variance. Logistic regression analysis was performed, and odds ratios were calculated (95% confidence interval) to estimate the crushability of test foods (dependent variable). Active site, presence or absence of occlusal support, MMSE scores, and tongue pressure values were selected as independent variables. The *t*-test was used to compare tongue pressure values according to the crushability and the hardness of the test food. The χ^2^ test was used to compare the ratio of crushability. The cutoff value of the tongue pressure in relation to test food crushability by the tongue was assessed using the receiver operating characteristic curve. The significance level was defined as 5%.

## 3. Results

The participants included 72 (16 males, 56 females, mean age of 85.6 ± 6.6 years old) older adults with reduced cognitive function and requiring caregiving. Among them, 36 (50.0%) used dentures and 47 (65.3%) had occlusal support with residual teeth or artificial teeth. In addition, among 27 (37.5%) participants taking normal food, two participants ate without using dentures despite the loss of occlusal support (Table 2).

### 3.1. Test 1 (Free Crush)

The inter-rater agreement coefficient (95% CI) for active site assessment by two examiners was 0.97 (0.94–0.99), indicating good reliability. The active sites used to crush the food were consistent for both SJ and HJ, with 41 (56.9%) participants using “teeth–teeth,” 15 (20.8%) using “teeth–ridge,” 10 (13.9%) using “ridge–ridge,” and six (8.3%) using the “tongue” to crush the test food (Table 3). Among the six participants in whom the “tongue” was the active site, five had occlusal support from natural or artificial teeth. None of the six participants took normal food or presented with ambulatory ADL. Of the 10 participants with a “good” nutritional status according to the MNA-SF score, the active site was “teeth–teeth” in nine. We observed no significant difference in age, sex, food form, nutrition status, or the MMSE scores among the participants with different active sites, and the tongue pressure tended to be significantly lower in participants with “tongue” as the active site than in those with other active sites (Table 3).

Seventy participants (97.2%) crushed the SJ and 62 (86.1%) crushed the HJ by free crush. The active site in the two participants unable to crush the SJ was “tongue”. Meanwhile, all six participants with “tongue” as the active site could not crush the HJ (Table 4). Tongue pressure in participants that crushed the SJ by free crush was 18.6 ± 8.6 kPa; the corresponding values in those that could not crush the SJ were 4.3 kPa and 2.9 kPa. Tongue pressure values of participants who could and could not crush the HJ by free crush were 19.4 ± 8.3 kPa and 11.0 ± 8.7 kPa, respectively. Significant differences were observed in the tongue pressure according to the crushability of HJ (*p* < 0.01) (Table 4).

Furthermore, logistic regression analysis revealed significant associations between the crushability of HJ and active site variation (OR: 0.10, 95% CI: 0.02–0.48; *p* < 0.01) (Table 5).

### 3.2. Test 2 (Tongue Crush)

Sixty-four (88.9%) participants could crush the SJ with their tongue. Twenty-three (31.9%) participants crushed the HJ. Tongue pressure in participants that could crush the SJ with their tongue was 20.1 ± 7.5 kPa. The corresponding value in participants that could not crush the SJ with their tongue was 3.4 ± 1.4 kPa. Furthermore, the tongue pressure of participants who could and could not crush the HJ with their tongue was 26.8 ± 5.5 kPa and 14.2 ± 6.9 kPa, respectively (Table 6). Significant differences were observed in tongue pressure values according to the crushability and hardness of the test food.

**Figure 3:**
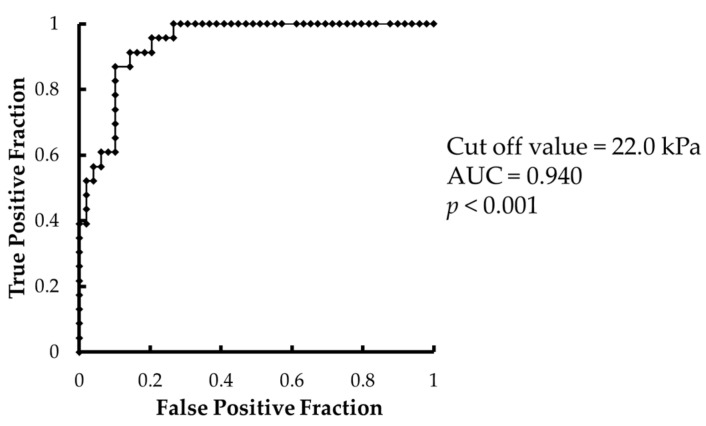
Receiver operating characteristic curve of tongue pressure versus crushability of the HJ with the tongue.

The minimum tongue pressure in participants that could crush the SJ with their tongue was 6.2 kPa, and the maximum tongue pressure in those that could not crush the SJ was 4.9 kPa. The area under the receiver operating characteristic curve of the crushability of HJ with tongue versus tongue pressure was 0.940 (*p* < 0.001), and the cutoff value of tongue pressure was 22.0 kPa (Figure 3). The cutoff value was obtained by the closest point from the upper left corner.

### 3.3. Comparison of Crushing Method and the Hardness of the Test Food

Significant differences were observed in the rate of crushability according to the hardness of the test food and crushing method. Furthermore, significant differences were observed in the tongue pressure of “crushable” according to the hardness of the test food on Test 2 and to the crushing method on the HJ (Table 7).

## 4. Discussion

This study aimed to establish a method of oral function assessment to determine food crushing capacity in older adults with cognitive function decline and requiring care, to inform food types that can be safely consumed by this group. We focused on older adults requiring caregiving who do not wear dentures despite losing the occlusal contact between the remaining teeth. The present findings may help inform care protocols that rely on individuals’ ability to crush food, eat, and swallow, and that may be affected by the site of food crushing.

In Japan, the number of older adults with dementia and care needs is rapidly increasing. The rate of denture usage among older adults with dementia is low [7]; dementia is a risk factor for choking [9,10]. Consequently, our study included older adults with cognitive decline requiring caregiving. However, we excluded older adults who could not understand the provided instructions. The MMSE scores suggest that the participants in this study had mild to moderate dementia. Many dementia patients do not wear dentures despite having lost occlusal contact [5,6,7]. In this study, 31.9% of the participants did not wear dentures despite having lost occlusal support. However, two of these participants were given normal food; both participants had a strong preference for normal food, which was provided to them at the discretion of their physician and nurses after obtaining consent from the participants and their families, taking into consideration the risk of choking given their relatively high level of cognitive function and independence with activities of daily living. As it has become clear that there are many older adults requiring caregiving who are capable of understanding simple instructions but do not wear dentures and that, albeit few, there are older adults taking normal food who do not wear dentures despite having lost occlusal support, we believe it is necessary to focus on the management of choking risks in such older adults requiring caregiving.

A total of 91.7% of the participants had active sites of “teeth–teeth,” “teeth–ridge,” or “ridge–ridge,” and these participants were crushing the test food by transferring the food to the molars and ridge and cycle of the mandible for mastication while keeping the food between their molars and ridge [33]. Only 8.3% of the participants used the “tongue” as the active site, suggesting that most people transfer the food to their molars or ridge and cycle of the mandible when trying to crush food freely. Meanwhile, five of 47 participants with occlusal support tried to crush the food using their tongue, which suggested that the active site is not necessarily related to the presence or absence of occlusal support. However, outcomes may differ among test food types, including those harder than jelly (e.g., a cookie); thus, the reported active sites may correspond to soft food consumption. A total of 25 of 27 participants given normal food used “teeth–teeth” as the active site, and none of those taking normal food used the “tongue” as the active site; our findings suggest that the decision to provide normal food for safe ingestion may be affected by whether or not the recipient can transfer the food and cycle of mandible on a daily basis.

In addition, although there was no ambulatory participant with “tongue” as the active site, the number of bedridden participants in this study was small. Hence, further studies are needed to examine the relationship between active sites and ADL. The active site for many of the participants with a “good” nutritional status was “teeth–teeth”. Remaining teeth and denture usage help maintain or improve the nutritional status [34,35,36]; our study suggests that the presence of occlusal support provided by the remaining or artificial teeth had a favorable impact on the nutritional status. However, as only nine of 41 participants who used “teeth–teeth” as the active site were considered to have a “good” nutritional status, our results could not confirm a direct causal relationship between the active site and nutritional status.

There was no relationship between the active site and cognitive function as there was no significant difference in the MMSE scores among the groups with different active sites. This finding may be attributed to the exclusion of individuals incapable of comprehending simple instructions.

In this study, tongue pressure values of participants using “tongue” as the active site were lower than those of participants using other parts of the mouth as the active site. Hiiemae et al. reported that food is placed on the dorsum of the tongue after the food is ingested; then, the entire tongue moves backward and downward with the opening of the mouth, and rotates outward to move the food to the occlusal surface of the mandible (pull-back movement) [37]. Our findings suggest that the participants with low tongue pressure may have reduced motor function of the tongue, including reduced ability to perform the pull-back movement and difficulty in transferring the food to the molars and ridge and keeping it there. When the participants were instructed to exert some force to crush the food, those with weakened oral function tended to crush the food with their tongue.

Meanwhile, Yokoyama et al. reported that the crushing of jelly using the tongue in younger persons required tongue pressure far greater than that required by chewing [38]. Tongue pressure in this study was measured with a sensor sheet attached to the participant’s palate while they were chewing. In addition, as the younger participants were instructed to chew, it is likely that they could transport the food to the molars. The tongue pressure measured in the present study is the maximum pressure measured using a balloon probe. Older adults that require caregiving and have reduced oral function, including maximum tongue pressure values, may have difficulty transporting food to the molars and moving the jaw while keeping the food at the molars.

Furthermore, Kojima et al. reported that semi-solids such as jelly are generally crushed by the tongue, and that chewing may be affected by the height of the palate [39]. However, the hardness of the jelly in that report was 4600 N/m², a lot softer than the jelly used in the present study, suggesting it was not a “tongue mashable” food according to the universal design food classifications. The discrepancy in testing conditions precludes meaningful comparisons between the present and previous studies.

In both the “free crush” and the “tongue crush” categories, fewer participants crushed the HJ, which was the harder test food, suggesting that food hardness affects its crushability. Furthermore, the results of our study suggest that in free crushing conditions, people who try to crush food between the tongue and palate are less likely to crush the food than those who try to crush the food with their teeth or ridges. Therefore, older adults with reduced cognitive function requiring caregiving and food support should be assessed for their ability to transfer food to the molars and ridge and cycle of the mandible while keeping the food between their molars or ridges, regardless of their dentition status. In addition, the active site used affects the crushability of HJ during a “free crush”. When crushing food as hard as soft rice, the involvement of the teeth or residual ridge may be relevant.

The crushability of food by tongue in this study was associated with tongue pressure. A tongue pressure of 6.2 kPa or higher was required to crush the SJ, and that of 22.0 kPa or higher was required to crush the HJ.

The fact that tongue pressure that allowed HJ to be crushed was higher in the “tongue crush” than in the “free crush” condition suggests that, if the food can be transported to the molars and crushed with the teeth or residual ridge, it would not require the tongue pressure involved in crushing with the tongue. However, since food crushing, including chewing, requires the concerted action of several oral organs, tongue pressure alone may not determine crushability and the active site, and further studies are required to elucidate the other factors involved. The presented findings are based on a single test food type, precluding generalization of tongue pressure values to other contexts. Nevertheless, these findings provide preliminary insights into the relationship between the crushability of food by the tongue and tongue pressure values; further studies are required to understand the impact of food hardness in this context. The tongue pressure test could help predict if the food can be crushed using the tongue, and it may be helpful to select food that is easiest to crush in the mouth.

This study suggests that assessing tongue pressure and intraoral active site for crushing food could help determine an appropriate diet for older adults requiring nursing home care. Such assessments may support professionals in fields other than dentistry in selecting diets most suitable for patients in their care.

## 5. Conclusions

Assessments of tongue pressure and intraoral active sites for food crushing may help determine an appropriate diet for older adults requiring nursing home care.

## Figures and Tables

**Table 1:** Definition and classification of active site by visual examination.

Active Site	Mandibular Movement	Food Stay	How to Crush
Teeth–teeth	Cycle toward right or leftfor mastication	On teeth	By occlusion between maxillary and mandibular residual or artificial teeth
Teeth–ridge	Cycle toward right or left	On teeth or ridge	Between the maxillary or mandibular residual or artificial teeth and residual ridge in the opposing arch
Ridge–ridge	Cycle toward right or left	On ridge	Between the maxillary and mandibular ridges
Tongue	Cycle or up and down	On tongue	By pressing the test food between the tongue and palate

**Table 2:** Number of denture users, food forms, and remaining teeth according to the presence or absence of occlusal support (*n* = 72).

	Presence or Absence of Occlusal Support		Total	*p*-Value
	Present	Absent		
Denture usage				
Yes	31 (43.1%)	5 (6.9%)	36 (50.0%)	
No	16 (22.2%)	20 (27.8%)	36 (50.0%)	<0.001
Food form				
Normal food	25 (34.7%)	2 (2.8%)	27 (37.5%)	
Chopped food	18 (25.0%)	17 (23.6%)	35 (48.6%)	
Modified diet for dysphagia	4 (5.6%)	6 (8.3%)	10 (13.9%)	<0.001
Number of remaining teeth	13.1 ± 9.6	4.5 ± 4.5		<0.05
	47 (65.3%)	25 (34.7%)	72 (100%)	

**Table 3:** Participant characteristics per active site (*n* = 72).

	Teeth–Teeth	Teeth–Ridge	Ridge–Ridge	Tongue	Total
Age					
(years)	85.4 ± 6.6	86.7 ± 6.2	85.0 ± 4.9	85.3 ± 10.4	
Sex, *n* (%)					
(Male)	8 (11.1%)	4 (5.6%)	3 (4.2%)	1 (1.4%)	16 (22.2%)
(Female)	33 (45.8%)	11 (15.3%)	7 (9.7%)	5 (6.9%)	56 (77.8%)
Occlusal support					
Present	41 (56.9%)	1 (1.4%)	0	5 (6.9%)	47 (65.3%)
Absent	0	14 (19.4%)	10 (13.9%)	1 (1.4%)	25 (34.7%)
Food form					
Normal food	25 (34.7%)	1 (1.4%)	1 (1.4%)	0	27 (37.5%)
Chopped food	15 (20.8%)	10 (13.9%)	8 (11.1%)	2 (2.8%)	35 (48.6%)
Modified diet for dysphagia	1 (1.4%)	4 (5.6%)	1 (1.4%)	4 (5.6%)	10 (13.9%)
ADL *					
Ambulatory	21 (29.2%)	5 (6.9%)	5 (6.9%)	0	31 (43.1%)
Wheelchair use	19 (26.4%)	9 (12.5%)	5 (6.9%)	4 (5.6%)	37 (51.4%)
Bedridden	1 (1.4%)	1 (1.4%)	0	2 (2.8%)	4 (5.6%)
Nutrition status					
Good	9 (12.5%)	0	1 (1.4%)	0	10 (13.9%)
At risk	29 (40.3%)	11 (15.3%)	7 (9.7%)	3 (4.2%)	50 (69.4%)
Malnutrition	3 (4.2%)	4 (5.6%)	2 (2.8%)	3 (4.2%)	12 (16.7%)
MMSE					
(score)	18.2 ± 6.6	14.3 ± 5.5	13.4 ± 8.6	12.8 ± 7.0	
Tongue pressure **					
(kPa)	18.8 ± 8.1	20.8 ± 9.0	19.0 ± 8.8	6.4 ± 3.9	
	41 (56.9%)	15 (20.8%)	10 (13.9%)	6 (8.3%)	72 (100%)

* There were significant differences between teeth–teeth and tongue (*p* < 0.01), and between ridge–ridge and tongue (*p* < 0.05). ** There were significant differences between teeth–teeth and tongue (*p* < 0.01), between teeth–ridge and tongue (*p* < 0.01), and between ridge–ridge and tongue (*p* < 0.05).

**Table 4:** Crushability by free crush (*n* = 72).

	Crushable	Not Crushable	*p*-Value
SJ			
Participants, *n* (%)	70 (97.2%)	2 (2.8%)	
(Active site)			
Teeth–teeth	41 (56.9%)	0	
Teeth–ridge	15 (20.8%)	0	
Ridge–ridge	10 (13.9%)	0	
Tongue	4 (5.6%)	2 (2.8%)	
Tongue pressure (kPa)	18.6 ± 8.6	3.6 ± 1.0	<0.05
HJ			
Participants	62 (86.1%)	10 (13.9%)	
(Active site)			
Teeth–teeth	41 (56.9%)	0	
Teeth–ridge	12 (16.7%)	3 (4.2%)	
Ridge–ridge	9 (12.5%)	1 (1.4%)	
Tongue	0	6 (8.3%)	
Tongue pressure (kPa)	19.4 ± 8.3	11.0 ± 8.7	<0.01

**Table 5:** Logistic regression analysis of the crushability of HJ.

	OR	95% CI	*p*-Value
Active site	0.10	0.02–0.48	<0.01
Presence or absence of occlusal support	0.12	0.01–2.79	0.187
MMSE	1.08	0.90–1.29	0.389
Tongue pressure (kPa)	1.01	0.86–1.17	0.941

**Table 6:** Crushability by tongue crush (*n* = 72).

	**Crushable**	**Not Crushable**	***p*-Value**
SJ			
Participants, *n* (%)	64 (88.9%)	8 (11.1%)	
Tongue pressure (kPa)	20.1 ± 7.5	3.4 ± 1.4	<0.001
HJ			
Participants	23 (31.9%)	49 (68.1%)	
Tongue pressure (kPa)	26.8 ± 5.5	14.2 ± 6.9	<0.001

**Table 7:** Comparison of the rate of crushability and tongue pressure.

	Test 1 (Free Crush)		Test 2 (Tongue Crush)		*p*-Value
	Crushable	Not Crushable	Crushable	Not Crushable	
Participants					
SJ	70	2	64	8	0.0492
HJ	62	10	23	49	<0.001
*p*-value	0.0159		<0.001		
Tongue pressure (kPa)					
SJ	18.6 ± 8.6		20.1 ± 7.5		0.305
HJ	19.4 ± 8.3		26.8 ± 5.5		<0.001
*p*-value	0.615		<0.001		

## Data Availability

The data presented in this study are available on request from the corresponding author. The data are not publicly available due to privacy laws.
